# Serum Type I Interferon Score for Prediction of Clinically Meaningful Disease Progression in Limited Cutaneous Systemic Sclerosis

**DOI:** 10.1002/art.43120

**Published:** 2025-03-03

**Authors:** Stefano Di Donato, Rebecca Ross, Ranjitha Karanth, Vishal Kakkar, Enrico De Lorenzis, Lesley‐Anne Bissell, Kristina Clark, Philip Yee, Christopher P. Denton, Francesco Del Galdo

**Affiliations:** ^1^ Leeds Institute of Rheumatic and Musculoskeletal Medicine, University of Leeds and Leeds Biomedical Research Centre, National Institute for Health Research, Leeds Teaching Hospitals NHS Trust Leeds United Kingdom; ^2^ Leeds Institute of Rheumatic and Musculoskeletal Medicine, University of Leeds Leeds United Kingdom; ^3^ Catholic University of the Sacred Heart, Fondazione Policlinico Universitario Agostino Gemelli IRCCS Rome Italy; ^4^ University College London London United Kingdom

## Abstract

**Objective:**

To assess the value of serum type I interferon (IFN) score in predicting clinically meaningful progression in limited cutaneous systemic sclerosis (lcSSc) using a novel composite endpoint adopted from the MINIMISE clinical trial.

**Methods:**

A retrospective, longitudinal lcSSc cohort was identified within a national, multicenter observational cohort. The MINIMISE trial combined Morbi‐mortality endpoint was used as the clinical outcome for a time to clinical worsening (TTCW) design. The IFN score was calculated from the serum concentration of chemokine C‐C motif ligand (CCL) 2, CCL8, CCL19, C‐X‐C motif chemokine ligand (CXCL) 9, CXCL10, and CXCL11 on patients and age‐ and sex‐matched healthy controls (HCs). A “high” IFN score was defined as 2 SDs above the HC mean. The association of the IFN score with TTCW was assessed using Cox proportional hazard regressions and Kaplan‐Meier curves. The potential improvement in risk stratification when combining IFN score with lcSSc clinical features was explored.

**Results:**

A total of 149 patients were included in the analysis: 67 “high” IFN and 82 “low.” High IFN patients presented a shorter TTCW (74.7 months [95% confidence interval (CI) 70.1–79.3] vs 110.6 months [95% CI 107.2–114.0]; *P* < 0.001) and met the endpoint in higher proportion compared with low IFN (55% vs 12%; *P* < 0.001). A high IFN score conferred hazard ratio (HR) 5.5 (95% CI 2.7–11.3) for TTCW compared with low IFN, and IFN score as a continuous variable conferred HR 2.38 (95% CI 1.4–4.0) for TTCW independently of clinical variables. Pulmonary arterial hypertension, interstitial lung disease, digital ulcers, and modified Rodnan skin score were associated with TTCW. An exploratory analysis showed that these clinical features improve risk stratification over time in combination with IFN score.

**Conclusion:**

Serum assessment of type I IFN activity is a valuable predictor of clinically meaningful outcomes in lcSSc. The combination of serum IFN score with sentinel clinical features can improve stratification strategies in clinical trials and patient management.

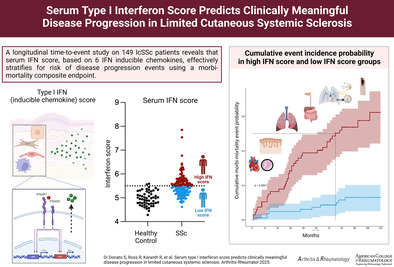

## INTRODUCTION

Patients with limited cutaneous systemic sclerosis (lcSSc) represent more than 60% of the SSc population and often experience a slower disease progression with reduced extent of fibrotic organ damage compared to patients within the diffuse cutaneous (dc) subset. Nonetheless, the lcSSc subset is still burdened by a considerable morbidity accrual over time, evident through various indicators such as esophageal dysfunction (62%), interstitial lung disease (ILD; 35%), digital ulcers (DUs; 37%), cardiac diastolic dysfunction (20%), and joint synovitis (13%).^1^, ^2^ Furthermore, lcSSc still carries a considerable overall mortality at 10 years of 22%,^3^ with 55% 3‐year mortality in patients developing pulmonary artery hypertension (PAH).^4^

The slower rate of progression in this subset and the need to observe and measure the efficacy of investigational drugs within a constrained timeframe of a clinical trial have led to a deep underrepresentation of patients with lcSSc in intervention studies, defining a highly unmet need in the field.^5^ Time‐to‐event trials allow the combination of multiple events, all defined as endpoints, and have been used successfully to measure the efficacy of interventions in complex populations with challenging outcome measures, including important examples of connective tissue disease‐associated PAH.^6^ The analysis of the Royal Free cohort of patients with lcSSc has led to the identification of clinically significant events in patients with lcSSc and their relative accrual over time.^7^ Building on this analysis, Denton et al have proposed the design of an intervention study for mycophenolate mofetil (MMF) employing a time‐to‐combined event endpoint (MINIMISE trial; EudraCT: 2019‐004139‐21; ClinicalTrials.gov: NCT04927390).^8^ The events considered in the MINIMISE combined endpoint included SSc‐related mortality and SSc‐related organ manifestations, such as new onset of PAH, new ILD or ILD progression, clinically significant worsening of the modified Rodnan skin score (mRSS), new onset of SSc‐related cardiac disease, new DUs requiring hospitalization, and significant gastrointestinal (GI) tract disease.

The composite “Morbi‐mortality” MINIMISE trial endpoint of “time to clinical worsening” (TTCW) reflected expert consensus of the most clinically relevant events that would be robustly captured during follow‐up in a clinical trial or observational cohort and that might reasonably be expected to be influenced by immunosuppressive disease‐modifying therapy such as MMF. Content validity is supported by several component endpoints showing reduced frequency in the Royal Free SSc Cohort in dcSSc from 2000 to 2003, when MMF was generally used for treating skin disease, compared with 1990 to 1993, when MMF was not routinely available.^9^ Type I interferon (IFN) activation has been linked to several aspects of SSc disease progression,^10^ including skin involvement,^11^, ^12^, ^13^ ILD,^14^, ^15^ DUs,^16^ and PAH.^17^ In this context, given that all these aspects are included in the MINIMISE endpoint, we aimed to analyze the TTCW of patients with lcSSc in a single‐center observational study and to determine the value of type I IFN activation in identifying risk strata for TTCW accrual over time.

Bauer et al identified that several chemokines, known to be induced in vitro by type I IFN, were most significantly up‐regulated in the serum of patients with systemic lupus erythematosus with high IFN signature as measured by peripheral blood transcriptome.^18^ In their work they indicated that the top six chemokines for concentration and significance were chemokine C‐C motif ligand (CCL) 2, also referred to as monocyte chemoattractant protein 1; CCL8, also known as monocyte chemoattractant protein 2; CCL19, also known as macrophage inflammatory protein‐3β; C‐X‐C motif chemokine ligand (CXCL) 10, also known as IFNγ‐induced protein 10; CXCL9, also known as IFNγ‐induced protein 9; and CXCL11, also known as IFNγ‐induced protein 11. Significantly, previous data highlighted that a score based on these six chemokines effectively stratifies clinical outcomes in dcSSc.^19^ Building upon this foundation, we developed a TTCW analysis to tailor this stratification approach to lcSSc, considering its distinct natural history.

## PATIENTS AND METHODS

### Study design and patients

The study was designed as a longitudinal retrospective study of incident patients aged ≥18 years fulfilling the 2013 ACR/EULAR classification criteria for SSc^20^ and presenting with the lcSSc subset as defined by LeRoy et al.^21^ All patients were enrolled from a single‐center observational cohort attending the Leeds Teaching Hospital Trust Rheumatology outpatient from December 1, 2013, until November 30, 2019. All patients provided informed consent, and the research was undertaken in compliance with the Declaration of Helsinki. Study protocol and relative informed consent were approved by the Human Research Ethics committee (“Leeds Teaching Hospitals Connective Tissue Disease and Vasculitis Cohort Cross‐sectional and Longitudinal Clinical and Basic Science Evaluation (CONVAS),” Research Ethics Committee reference: RR10/9608 from December 1, 2013, until January 1, 2015; and “Stratification for Risk of Progression in Systemic Sclerosis (STRIKE),” Research Ethics Committee reference 15/NE/0211, ID 178638 from January 1, 2015, to ongoing). Patients or the public were not involved in the design, conduct, reporting, and dissemination plans of this research.

The characterization at baseline of patients included sex, age, antinuclear antibody (ANA) positivity, antitopoisomerase I (Scl70) and anticentromere (ACA) positivity, pharmacologic treatment, disease duration defined from the first non–Raynaud Phenomenon SSc symptom, history or presence of DUs (referred as DU disease), presence of PAH, presence of calcinosis, mRSS, symptomatic gastroesophageal reflux disease, diagnosis of ILD, forced vital capacity (FVC), and diffusing capacity of the lungs for carbon monoxide (DLco) percent predicted. All patients received at least one follow‐up every 6 months until event development or censoring. Database extract and chart review were employed for the identification of clinically meaningful events qualifying for the study endpoint and relative clinical validation, when needed.

### IFN score calculations and threshold definition

The IFN score was calculated as the average of the natural logarithm of the concentration of CCL2, CCL8, CCL19, CXCL10, CXCL9, and CXCL11 based on the work from Bauer et al.^18^ Using the methodology previously established in dcSSc, the threshold for the definition of a “high” IFN score was set as the mean of the IFN score calculated on 72 healthy volunteers matched for sex and age (data not shown) plus 2 standard deviations (SDs).^19^

### Objective of the study and endpoint definition

The objective of the study was to explore the value of IFN score in predicting the risk of clinically significant events over time in patients affected by lcSSc and enrolled in our observational cohort. Patients were included in the analysis if they fulfilled the 2013 ACR/EULAR classification criteria for SSc within the lcSSc subset at baseline and had an available serum sample within 3 months from the baseline, with at least 48 months of observation.

For the assessment of the MINIMISE endpoint, we analyzed the cohort for the occurrence of the first of the following “Morbi‐mortality” events: SSc‐related death, new diagnosis or progressive lung fibrosis as defined by an absolute FVC% drop of ≥10% in 12 months or between 5% and 9% with a 15% DLco reduction, new right heart catheter‐diagnosed PAH according to the current definition at the time of the diagnosis, new onset of scleroderma renal crisis, new onset of major cardiac complication related to SSc as defined by systolic ejection fraction drop to <45% or pericardial effusion impairing cardiac function or arrhythmia requiring antiarrhythmic therapy (cardioversion or medical) or need for a cardiac device, new onset of SSc‐related GI disease requiring enteral nutrition supplementation for at least 3 weeks or any parenteral feeding or hospital admission for intestinal obstruction or pseudo‐obstruction, severe digital vasculopathy requiring hospitalization (including gangrene, amputation, osteomyelitis, or septic arthritis, and excluding elective hospitalization for vasodilator treatment), and an mRSS absolute increase of ≥5 units and a 25% increase from baseline (Supplementary File 1).

### Power analysis and sample size considerations

To inform an effect size for our event‐free survival analysis sample calculation, we used previous data from a large longitudinal SSc cohort that showed a combined cumulative incidence of clinically meaningful significant events (namely ILD, PAH, cardiac SSc involvement, and mRSS worsening) of approximately 40% over a period of 5 years in patients with lcSSc and of approximately 73% in patients with dcSSc.^7^ Prior analysis indicated that 50% of patients had a high IFN score; hence, we set a ratio between the IFN high and IFN low arms of 1 to 1. We hypothesized a hazard ratio (HR) for IFN high versus IFN low of 2.0. For this analysis, we set a maximum accrual duration of 10 years, a power of 80%, and an alpha of 5%, all with a two‐sided hypothesis test. Using the R package ‘gsDesign,’ a sample size of 56 patients for each group and 42 required events were needed. Adjusting the overall 112 patients required for a censoring up to 25%, we set 140 patients as the minimum sample size for our study. Based on these assumptions, we analyzed the serum IFN score on the first 150 consecutive patients with lcSSc enrolled in the observational study with at least 4 years of observation.

### Statistical analysis

Categorical data were reported as counts and percentages and comparisons of categorical data were performed using Fisher exact test or Pearson chi‐square test, as appropriate. Continuous data were reported as mean (SD) or medians (interquartile range [IQR]), and normality was assessed with the Shapiro‐Wilk significance test, with a graphical check through density plots and QQ plots. The homogeneity of variance for continuous variables was assessed using an F test, and comparisons of continuous data were performed with Student's *t*‐test or Wilcoxon test, as appropriate. The date of serum sample collection was considered as the formal baseline in the time‐to‐event analysis. Event‐free survival for IFN groups was plotted using Kaplan‐Meier (KM) curves, and the comparison between the two restricted mean TTCW was computed non‐parametrically as the area under the KM curve, using the smaller of the maximum observed times in the two groups. Confidence intervals (CIs) and statistical significance for the comparisons were derived using the Greenwood plug‐in estimator for variance. The event‐free survival probabilities across the two groups were compared at three timepoints potentially relevant for clinical trial design and clinical management (12, 24, and 36 months) using the difference in KM estimates of survival. Because SSc‐related death was included in the composite Morbi‐mortality event, we did not need to account for this as a competing risk before clinical worsening.

To investigate the association between IFN score and the risk over time of meeting the endpoint and to account for known disease domains that prognostically affect the development of disease complications included in the endpoint, univariable and multivariable Cox proportional hazard (CPH) regressions were performed. To account for potential nonlinear relationships between the IFN score and the outcome, we employed a penalized spline function when modeling the IFN score as a continuous variable. The proportional hazards assumption was tested via complementary log‐log plot. To visually assess the multivariable Cox regressions using the IFN score, we chose time‐dependent receiver operating characteristic (ROC) curves that change over time to provide a fuller description of the models.^22^, ^23^ The event‐free survival prediction accuracy of the models was assessed using bootstrap resampling with 1,000 iterations. We then calculated the concordance index (C‐index) and area under the curve (AUC) of the models at three timepoints (12 months, 24 months, and 36 months).

The relationship between clinical features and IFN score on patient outcomes was explored using both visual and statistical methods. Clinical variables such as mRSS, PAH, ILD, DUs, and IFN score were dichotomized. UpSet plots were employed to visualize the intersections of various clinical and IFN score combinations and their associated event rates. KM curves were generated for newly identified high‐risk and low‐risk groups, with survival differences assessed using the log‐rank test. Additionally, a forest plot was used to compare the relative risks (RRs) of different feature combinations over multiple time points, offering a clear comparison between high‐risk and low‐risk groups. Data analysis was conducted using R core team software (RStudio) and the packages ‘anytime,’ ‘survival,’ ‘survminer,’ ‘ComparisonSurv,’ ‘survRM2,’ ‘ggsurvfit,’ ‘gtsummary,’ ‘moonBook,’ ‘survivalROC,’ ‘risksetROC,’ and ‘ggplot2.’

## RESULTS

Complete data were available for 149 patients, 143 (96%) of whom were women. The mean ± SD age at baseline was 60 ± 16 years, and the median (IQR) disease duration from the first non–Raynaud Phenomenon symptom was 8 (IQR 10) years. A total of 141 (95%) patients were ANA positive, 100 (67%) were ACA positive, 12 (8.1%) were Scl70 positive, and 35 (23%) patients were anti‐Ro52 positive. At baseline, 36 (24%) patients had ILD, 58 (39%) had DU disease, 53 (36%) had calcinosis, 9 (6%) had right heart catheter‐diagnosed PAH, and 127 (85%) were receiving proton pump inhibitor treatment for upper GI involvement. Mean ± SD mRSS was 2.09 ± 2.63, the mean ± SD FVC% was 109% ± 20%, and the mean ± SD DLco% was 67% ± 15%. Of the nine patients with PAH at baseline, six were receiving combination therapy (four with phosphodiesterase 5 inhibitors [PDE5is] combined with endothelin receptor antagonists [ERAs], one with calcium channel blockers combined with PDE5is and ERAs, and one with angiotensin‐converting enzyme inhibitors combined with PDE5is) and three single therapy (two receiving ERAs and one receiving intravenous iloprost) based on patients’ tolerability and in line with the current standard of care in the United Kingdom at the time of the analysis. The remaining treatments are reported in Table 1.

**Table 1:** Overall cohort demographic and clinical characteristics divided by event onset during follow‐up*

Characteristic		Cohort by event				Cohort by IFN group			
	Overall (N = 149)	No event (n = 102)	Event (n = 47)	*P* value^a^	*Q* value^b^	IFN low (n = 82)	IFN high (n = 67)	*P* value^a^	*Q* value^b^
Age, mean (SD), y	60 (16)	60 (17)	64 (16)	0.035	0.087	58 (15)	66 (16)	0.002	0.013
Sex, n (%)				0.4	0.6			0.4	0.6
Female	143 (96)	99 (97)	44 (94)	–	–	80 (98)	63 (94)	–	–
Male	6 (4.0)	3 (2.9)	3 (6.4)	–	–	2 (2.4)	4 (6.0)	–	–
Disease duration, median (IQR), y	8 (10)	8 (10)	8 (11)	0.9	>0.9	8 (10)	5 (9)	0.4	0.6
ANA positive, n (%)	141 (95)	96 (94)	45 (96)	>0.9	>0.9	76 (93)	65 (97)	0.3	0.6
ACAs, n (%)	100 (67)	70 (69)	30 (64)	0.6	0.8	56 (68)	44 (66)	0.7	0.8
Isolated ACA positivity, n (%)				0.009	0.052			0.086	0.4
ACA only	59 (40)	47 (46)	12 (26)	–	–	37 (45)	22 (33)	–	–
Non‐ACA only	90 (60)	55 (54)	35 (74)	–	–	45 (55)	45 (67)	–	–
Anti topoisomerase I antibodies, n (%)	12 (8.1)	8 (7.8)	4 (8.5)	>0.9	>0.9	8 (9.8)	4 (6.0)	0.4	0.6
Anti‐Ro52 antibodies, n (%)	35 (23)	20 (20)	15 (32)	0.1	0.2	12 (15)	23 (34)	0.005	0.027
Anti‐RNA polymerase III antibodies, n (%)	4 (2.7)	2 (2.0)	2 (4.3)	0.6	0.8	1 (1.2)	3 (4.5)	0.3	0.6
Anti U1‐RNP1 antibodies, n (%)	8 (5.4)	6 (5.5)	2 (4.3)	>0.9	>0.9	3 (3.7)	5 (7.5)	0.5	0.6
mRSS, mean (SD)	2.09 (2.63)	1.59 (2.05)	3.17 (3.36)	<0.001	0.005	1.93 (2.15)	2.28 (3.12)	0.7	0.9
FVC, median (IQR), %	109 (20)	113 (18)	100 (21)	<0.001	0.005	109 (20)	108 (20)	0.5	0.6
DLco, median (IQR), %	67 (15)	72 (14)	58 (13)	<0.001	<0.001	69 (15)	64 (14)	0.028	0.13
Upper gastrointestinal symptoms, n (%)	96 (64)	62 (61)	34 (72)	0.2	0.3	51 (62)	45 (67)	0.5	0.6
Calcinosis, n (%)	53 (36)	35 (34)	18 (38)	0.6	0.8	27 (33)	26 (39)	0.5	0.6
Interstitial lung disease, n (%)	36 (24)	17 (17)	19 (40)	0.002	0.007	18 (22)	18 (27)	0.5	0.6
Digital ulcers disease, n (%)	58 (39)	33 (32)	25 (53)	0.015	0.052	30 (37)	28 (42)	0.5	0.6
Pulmonary artery hypertension, n (%)	9 (6.0)	2 (2.0)	7 (15)	0.005	0.018	3 (3.7)	6 (9.0)	0.3	0.6
Sjögren syndrome overlap, n (%)	15 (10)	11 (11)	4 (8.5)	0.8	>0.9	6 (7.3)	9 (13)	0.2	0.6
Mycophenolate mofetil, n (%)	13 (8.7)	9 (8.8)	4 (8.5)	>0.9	>0.9	9 (11)	4 (6.0)	0.3	0.6
Aspirin, n (%)	30 (20)	20 (20)	10 (21)	0.8	>0.9	15 (18)	15 (22)	0.5	0.6
Calcium channel blockers, n (%)	94 (63)	67 (66)	27 (57)	0.3	0.6	54 (66)	40 (60)	0.4	0.6
Hydroxychloroquine, n (%)	26 (17)	19 (19)	7 (15)	0.6	0.7	17 (21)	9 (13)	0.2	0.6
Endothelin receptor antagonists, n (%)	7 (4.7)	2 (2.0)	5 (11)	0.032	0.087	3 (3.7)	4 (6.0)	0.7	0.8
Phosphodiesterase 5 inhibitors, n (%)	24 (16)	14 (14)	10 (21)	0.2	0.4	16 (20)	8 (12)	0.2	0.6
Intravenous iloprost, n (%)	26 (17)	14 (14)	12 (26)	0.078	0.2	16 (20)	10 (15)	0.5	0.6
ACE inhibitors, n (%)	51 (34)	33 (32)	18 (38)	0.5	0.7	23 (28)	28 (42)	0.079	0.3
IFN score, mean (SD)	5.45 (0.46)	5.34 (0.38)	5.67 (0.37)	<0.001	<0.001	5.24 (0.27)	5.70 (0.34)	<0.001	<0.001
Event, n (%)								<0.001	<0.001
Cardiac event	6 (4.0)	–	6 (13)	–	–	2 (2.4)	4 (6.0)		
Gastrointestinal failure	4 (2.7)	–	4 (8.5)	–	–	0 (0)	4 (6.0)		
SSc‐related mortality	9 (6.0)	–	9 (19)	–	–	1 (1.2)	8 (12)		
No event	102 (68)	102 (100)	–	–	–	72 (88)	30 (45)		
Pulmonary artery hypertension	13 (8.7)	–	13 (28)	–	–	4 (4.9)	9 (13)		
Interstitial lung disease progression	6 (4.0)	–	6 (13)	–	–	2 (2.4)	4 (6.0)		
Renal crisis	1 (0.7)	–	1 (2.1)	–	–	0 (0)	1 (1.5)		
Severe digital vasculopathy	3 (2.0)	–	3 (6.4)	–	–	1 (1.2)	2 (3.0)		
Skin worsening (mRSS)	5 (3.4)	–	5 (11)	–	–	0 (0)	5 (7.5)		

* ACA, anticentromere antibody; ACE, angiotensin‐converting enzyme; ANA, antinuclear antibody; DLco, diffusing capacity of the lungs for carbon monoxide; FVC, forced vital capacity; IFN, interferon; IQR, interquartile range; mRSS, modified Rodnan skin score; SSc, systemic sclerosis.

^a^ Wilcoxon rank sum test, Fisher exact test, or Pearson chi‐square test.

^b^ False discovery rate correction for multiple testing.

### Event accrual clinical analysis

The study was structured to ensure a minimum intended follow‐up duration of 4 years for each participant, extending up to a maximum of 10 years. The consecutive patients planned were recruited between December 2013 and December 2019; hence, we completed the 4‐year follow‐up of the last recruited patient in December 2023. The median (IQR) follow‐up of the cohort was 88 (IQR 48) months. A total of 77 patients were censored before 10 years follow‐up with a median (IQR) censoring time of 89 (IQR 9) months. A total of 47 (32.5%) patients developed the Morbi‐mortality endpoint by the time of the analysis with a restricted mean time to event of 94.5 (95% CI 78.4–111.3) months. The most common event was represented by new diagnosis of PAH (n = 13; 27.3%), followed by SSc‐related mortality (n = 9; 19%), progression of ILD (n = 6; 13%), SSc‐related cardiac events (n = 6; 13%), mRSS worsening (n = 5; 11%), GI complications (n = 4; 8.5%), severe digital vasculopathy requiring hospitalization (n = 3; 6.4%), and scleroderma renal crisis (n = 1; 2.1%).

**Figure 1:**
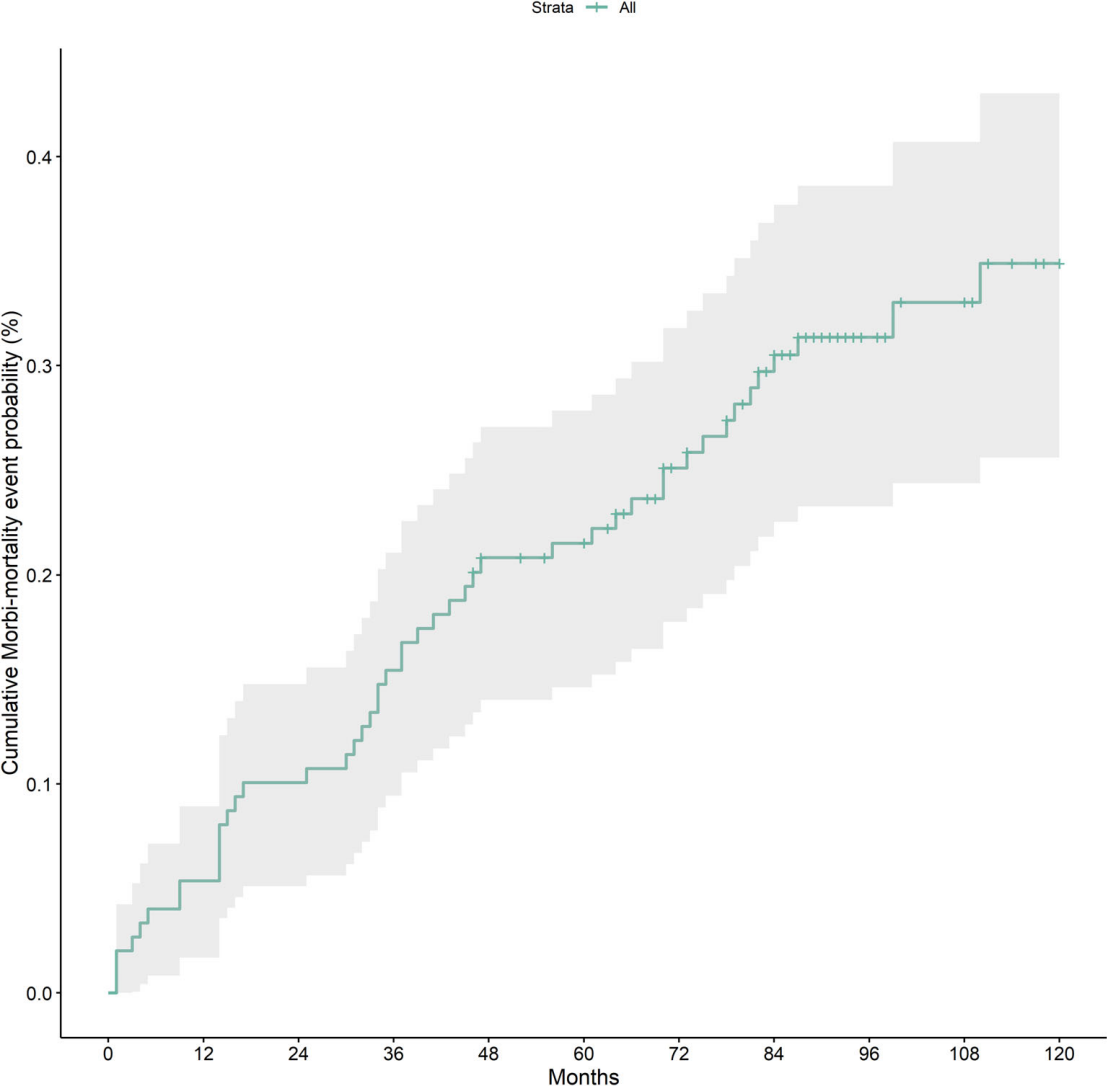
Kaplan‐Meier curve showing the overall cohort cumulative time to clinical worsening incidence probability. Color figure can be viewed in the online issue, which is available at http://onlinelibrary.wiley.com/doi/10.1002/art.43120/abstract.

Participants were censored at the time of first event. Subsequent events are reported in the Supplementary Material by IFN score group (Supplementary Table 1). The KM analysis for the overall cohort showed an event probability of 5.4% (95% CI 1.7%–8.9%) at 12 months, 10.1% (95% CI 5.1%–14.8%) at 24 months, 15.4% (95% CI 9.4%–21.0%) at 36 months, 21.5% (95% CI 14.6%–27.8%) at 60 months, and 34.9% (95% CI 25.6%–43.0%) at 120 months (Figure 1; Supplementary Table 2).

Patients who did not develop an event of interest had a significantly lower mean ± SD age at baseline compared with those who did (60 ± 17 vs 64 ± 16; *P* = 0.035) and a tendency toward higher prevalence of isolated ACA positivity (46% vs 26%; *P* = 0.053). Conversely, patients who developed the Morbi‐mortality endpoint presented a higher baseline mean ± SD mRSS (3.2 ± 3.4 vs 1.6 ± 2.1; *P* < 0.001) and a higher prevalence at baseline of DU disease (53% vs 32%; *P* = 0.015), ILD (40% vs 17%; *P* = 0.002), and PAH (15% vs 2%; *P* = 0.005). Accordingly, they also had a significantly lower mean ± SD FVC (100% ± 21% vs 113% ± 18%; *P* < 0.001), and DLco percentage predicted (58% ± 13% vs 72% ± 14%; *P* < 0.001).

### IFN score analysis

**Figure 2:**
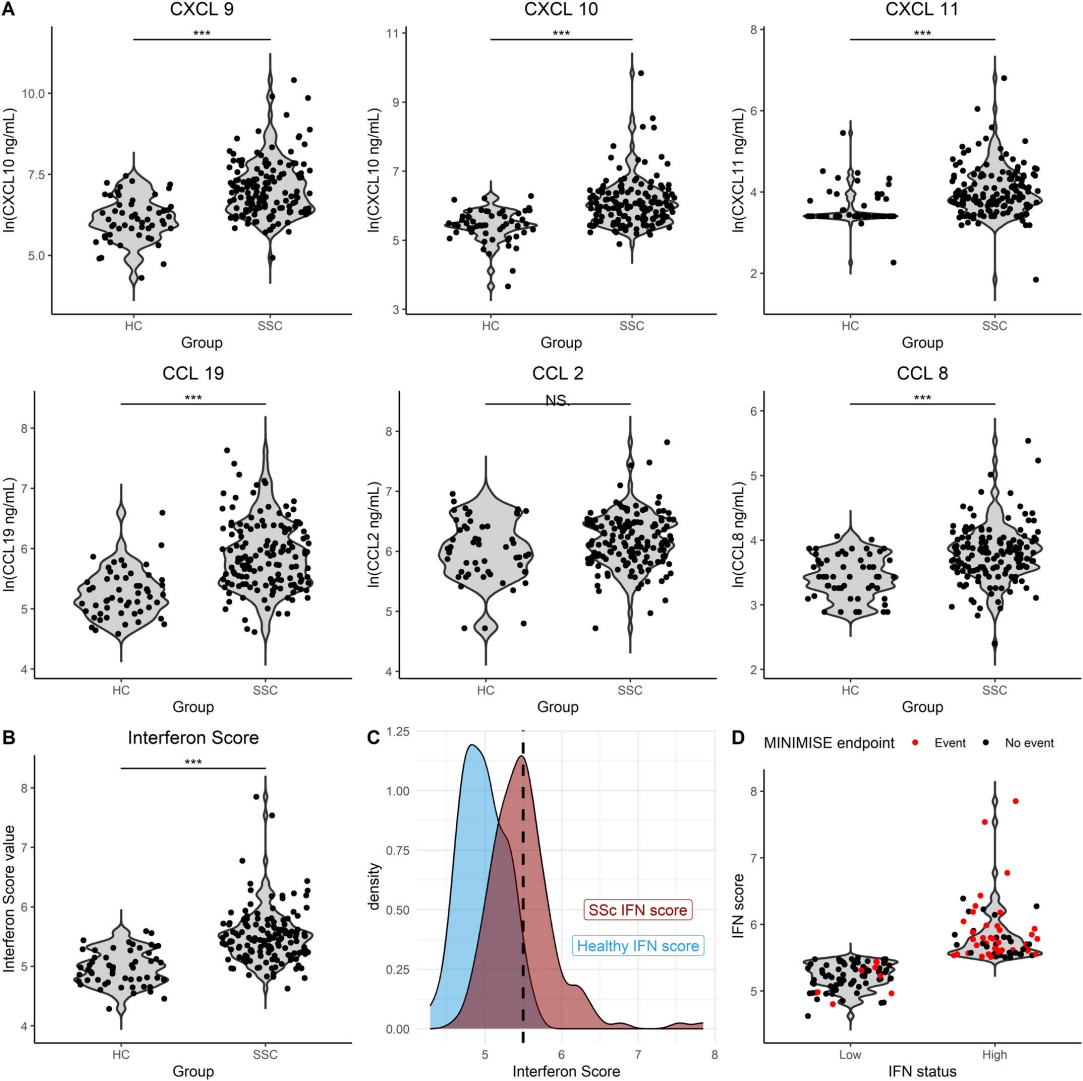
Type I IFN serum score in limited cutaneous SSc. (A) The concentration of the serum chemokines included in the IFN score was significantly higher in the limited cutaneous SSc cohort compared with a reference HC cohort matched for age and sex, except for CCL2 (Student's *t*‐tests with Bonferroni correction). (B) Violin plots with jittering showing the values of IFN scores of HCs and patients with SSc (Student's *t*‐test). (C) Density distributions of the IFN scores of HCs are in light blue and patients with SSc are in red. The dotted line represents the +2 SDs threshold from HC mean marking the IFN “high” and “low” categories. (D) Violin plots with jittering representing the individual IFN score values in the low (left pane) and high (right pane) groups; red dots represent the IFN scores of patients experiencing the outcome during follow‐up. ***P < 0.001. CCL, chemokine C‐C motif ligand; CXCL, C‐X‐C motif chemokine ligand; HC, healthy control; IFN, interferon; NS, not significant; SSc, systemic sclerosis.

The concentration of the serum chemokines included in the IFN score was significantly higher in the lcSSc cohort compared with a reference healthy control (HC) cohort matched for age and sex. Specifically, the natural logarithm (ln) of CXCL9 serum concentration was 6.93 ± 1.09 pg/mL (vs 6.11 ± 0.79 pg/mL; *P* < 0.001), ln CXCL10 was 5.99 ± 0.77 pg/mL (vs 5.43 ± 0.47 pg/mL; *P* < 0.001), ln CXCL11 was 3.89 ± 0.71 pg/mL (vs 3.40 ± 0.32 pg/mL; *P* < 0.001), ln CCL8 was 3.78 ± 0.46 pg/mL (vs 3.43 ± 0.43 pg/mL; *P* < 0.001), and ln CCL19 was 5.82 ± 0.83 pg/mL (vs 5.20 ± 0.53 pg/mL; *P* < 0.001). A numerical but not statistically significant difference was also observed for ln CCL2 (6.14 ± 0.57 pg/mL vs 6.00 ± 0.75 pg/mL) (Figure 2A). The IFN score presented a normal distribution in the lcSSc cohort with a mean ± SD value of 5.50 ± 0.44, which was significantly higher than that of the HCs (4.97 ± 0.27; *P* < 0.001) (Figure 2B). Sixty‐seven (45%) patients had an IFN score above two upper SDs of the matched HC mean (5.50) and were classified as high, whereas 82 (55%) were below this boundary and classified as “low” (Figure 2C). The distribution of the baseline clinical features across IFN groups is shown in Table 2.

**Table 2:** Univariable Cox proportional hazard regressions for Morbi‐mortality event occurrence*

Characteristic	HR	95% CI	*P* value	*Q* value^a^
Age	1.03	1.01–1.06	0.02	0.044
Sex				
Female	–	–	–	–
Male	1.50	0.47–4.84	0.5	0.7
Anticentromere	0.84	0.46–1.52	0.6	0.7
Antitopoisomerase I	1.03	0.37–2.88	>0.9	>0.9
Anti‐Ro52	1.51	0.82–2.79	0.2	0.3
Disease duration	1.00	0.97–1.04	0.8	>0.9
Isolated ACA positivity				
ACA only	–	–	–	–
Non‐ACA only	2.01	1.04–3.88	0.037	0.073
mRSS	1.13	1.05–1.21	<0.001	0.002
FVC	0.98	0.96–0.99	<0.001	0.002
DLco	0.94	0.92–0.96	<0.001	<0.001
Upper GI	1.43	0.75–2.70	0.3	0.4
Interstitial lung disease	2.73	1.52–4.89	<0.001	0.002
Digital ulcers disease	2.12	1.20–3.77	0.01	0.026
Calcinosis	1.19	0.66–2.14	0.6	0.7
Pulmonary artery hypertension	4.78	2.12–10.8	<0.001	0.001
Mycophenolate use	0.94	0.34–2.62	>0.9	>0.9
ET1 inhibitor use	5.45	2.10–14.2	<0.001	0.002
Interferon score			<0.001	<0.001
Linear component	2.82	1.58–4.69	<0.001	–
Nonlinear component	–	–	0.008	–
Interferon score group				
Low	–	–	–	–
High	6.20	3.07–12.5	<0.001	<0.001

* ACA, anticentromere antibody; CI, confidence interval; DLco, diffusing capacity of the lungs for carbon monoxide; ET1, endothelin 1; FVC, forced vital capacity; GI, gastrointestinal; HR, hazard ratio; mRSS, modified Rodnan skin score.

^a^ False discovery rate correction for multiple testing.

Patients within the IFN high group presented a greater incidence of Morbi‐mortality events compared with those in the IFN low (37 [55%] vs 10 [12%]; *P* < 0.001) (Figure 2D). IFN high patients had a higher median (IQR) age compared with IFN low patients (66 [IQR 16] vs 58 [IQR 15]; *P* = 0.01) and had a significantly higher prevalence of anti‐Ro52 antibodies (34% vs 15%; *P* = 0.025). No statistically significant difference was found across the two groups in terms of median (IQR) disease duration (8 [IQR 8] vs 5 [IQR 9]; *P* = 0.39), sex distribution, ethnicity, ANA positivity, ACA, antitopoisomerase I antibody status, and treatments (Table 1). Similarly, no statistically significant differences were present between the two subgroups for baseline organ‐specific manifestations, namely, ILD, DU disease, skin calcinosis history, PAH, mRSS, FVC%, DLco%, and upper GI involvement (Table 1).

### Parameters associated with the risk of event over time

At univariable CPH regression, the baseline clinical and demographics characteristics associated with an increased hazard of developing the endpoint over time were presence of PAH (HR 4.78, 95% CI 2.12–10.80; *P* = 0.001), presence of ILD (HR 2.73, 95% CI 1.52–4.89; *P* = 0.002), presence of DU disease (HR 2.12, 95% CI 1.20–3.77; *P* = 0.026), mRSS (HR 1.13, 95% CI 1.05–1.21; *P* = 0.002), age (HR 1.03, 95% CI 1.01–1.06; *P* = 0.044), FVC% (HR 0.98, 95% CI 0.96–0.99; *P* = 0.002), DLco% (HR 0.94, 95% CI 0.92–0.96; *P* < 0.001), and use of ERAs (HR 5.45, 95% CI 2.10–14.20; *P* = 0.002). In this context, IFN score, modeled using a penalized spline, was significantly associated with an increased hazard of developing the endpoint over time. The linear component showed a strong association (HR 2.82, 95% CI 1.58–4.69; *P* < 0.001), and its nonlinear component also contributed significantly (*P* = 0.008). When using a dichotomous model, being in the IFN high group at baseline conferred more than six‐fold hazard of Morbi‐mortality events over time compared with being in the IFN low group (HR 6.20, 95% CI 3.07–12.5; *P* < 0.001) (Table 2).

A total of 37 deaths occurred during follow‐up, 16 of which were directly attributed to scleroderma. Of these, 9 were included in the TTCW analysis as the first event for the patients, whereas the remaining 7 occurred after other outcome events. After adjusting for age, IFN score was not significantly associated with overall survival in the CPH model, as expected when most of the deaths were not related to SSc. In contrast, age emerged as a significant predictor of all‐cause mortality, with an HR of 1.16 (*P* < 0.001) (data not shown). Conversely, when considering deaths attributed to scleroderma (n = 16), after adjusting for age, IFN score had a significant positive association with SSc mortality in its linear component (HR 2.83, 95% CI 1.18–6.75; *P* = 0.019), whereas the nonlinear component did not reach statistical significance (*P* = 0.230). Age was also independently associated with SSc mortality (HR 1.12, 95% CI 1.04–1.20; *P* = 0.001).

A multivariable CPH regression model was built to test for the independence of the associations found at univariable analysis. We built two multivariable CPH models using the IFN score either as a continuous or a categorical variable, along with the other clinical features significantly associated with event accrual, namely mRSS, presence of baseline ILD, FVC, presence of baseline PAH, DLco, use of ERAs, DU disease, and age. Notably, informed by clinical plausibility and known collinearity, we opted to exclude DLco and FVC from our analysis, given their significant overlap with PAH and ILD, respectively, as well as the use of ERA because of its indication in DU disease and PAH. All the clinical features except for age retained statistical significance in both models, and IFN score was independently associated with an increased risk of events as both a continuous and a dichotomous variable (Table 3).

**Table 3:** Multivariate Cox proportional hazard regression models for IFN score as a continuous and categorical variable correcting for significantly associated clinical predictors of event in univariate analysis*

Characteristic	Model 1: IFN continuous			Model 2: IFN categorical		
	HR	95% CI	*P* value	HR	95% CI	*P* value
ILD	3.10	1.68–5.75	<0.001	3.13	1.69–5.77	<0.001
PAH	3.81	1.60–9.08	0.003	3.15	1.29–7.69	0.012
mRSS	1.14	1.04–1.25	0.006	1.12	1.03–1.21	0.005
DUs disease	1.77	0.98–3.22	0.059	2.06	1.13–3.74	0.018
Age	1.02	1.00–1.05	0.10	1.03	1.00–1.06	0.043
IFN score						
Linear component	2.38	1.41–4.01	0.004	–	–	–
Nonlinear component	–	–	0.005	–	–	–
IFN score group						
Low	–	–	–	–	–	–
High	–	–	–	5.53	2.70–11.3	<0.001

* CI, confidence interval; DU, digital ulcer; HR, hazard ratio; IFN, interferon; ILD, interstitial lung disease; mRSS, modified Rodnan skin score; PAH, pulmonary artery hypertension.

### ROC curve analysis of models’ performance

The performance of the multivariable CPH models containing IFN score as a continuous or categorical variable was assessed at three different time points. A time‐dependent incident case/dynamic control ROC curve approach was chosen mapping the risk score against the presence or absence of the event over time. The two models including the presence of baseline ILD, presence of baseline PAH, baseline mRSS, age, and IFN score showed an excellent AUC for all timepoints. In particular, the model built using the continuous IFN score modeled as a penalized spline showed an AUC ± SE of 0.799 ± 0.028, 0.803 ± 0.028, and 0.801 ± 0.029 for 12 months, 24 months, and 36 months, respectively, and a C‐index ± SE of 0.806 ± 0.029, whereas the model built using the categorical IFN score showed an AUC ± SE of 0.798 ± 0.029, 0.804 ± 0.030, and 0.805 ± 0.029 for 12 months, 24 months, and 36 months, respectively, with a C‐index ± SE of 0.794 ± 0.030. For the same timepoints, an exploratory model built including only the baseline presence of ILD, baseline presence of PAH, mRSS, and age showed an AUC ± SE of 0.723 ± 0.036, 0.716 ± 0.034, and 0.716 ± 0.033, respectively, and a C‐index ± SE of 0.713 ± 0.040, whereas a model built with IFN score alone had an AUC ± SE of 0.728 ± 0.032, 0.730 ± 0.032, and 0.730 ± 0.032, respectively, with a C‐index ± SE of 0.733 ± 0.032 (Supplementary Figure 1).

### Event‐free survival analysis

Patients in the IFN low group had a significantly higher 10‐year event‐free survival probability compared to the IFN high group, with a restricted mean TTCW of 110.6 months (95% CI 107.2–114.0) versus 74.7 months (95% CI 70.1–79.3) in IFN high (*P* < 0.001) (Supplementary Figure 2). The event probability of the IFN low group was significantly lower compared with the IFN high group at 12 months (1.2% [95% CI 0.0%–3.6%] vs 10.4% [95% CI 2.8%–17.5%]; *P* = 0.018), 24 months (4.9% [95% CI 0.1%–9.4%] vs 16.4% [95% CI 7.1%–24.8%]; *P* = 0.024), 36 months (4.9% [95% CI 0.1%–9.4%] vs 28.4% [95% CI 16.7%–38.4%]; *P* < 0.001), 60 months (7.4% [95% CI 1.5%–12.9%] vs 38.8% [95% CI 26.0%–49.4%]; *P* < 0.001), and 120 months (13.0% [95% CI 5.1%–20.2%] vs 62.3% [95% CI 44.3%–74.5%]; *P* < 0.001) (Supplementary Table 3 and Supplementary Figure 2). No significant difference in the median (IQR) censoring time in months was detected across IFN high and low groups (89 [IQR 9] vs 89 [IQR 8], respectively, *P* = 0.866). To further account for the possible influence of censoring, a KM analysis based on cases who had at least 5‐year follow‐up without censoring was performed. To do so, we filtered only patients who either developed an event in the first 5 years of follow‐up or had an event‐free follow‐up of at least 5 years. Using this approach, 136 patients were included in a 5‐year KM analysis, of whom there were 59 in the IFN high and 77 in the IFN low groups. INF low patients maintained a higher restricted mean TTCW compared with IFN high patients (103.5 months [95% CI 97.4–110.2] vs 74.1 [95% CI 62.8–85.3]; *P* < 0.001) (Supplementary Table 4 and Supplementary Figure 3).

### Combination of clinical and IFN score parameters identifies subsets of patients with distinct clinical outcomes over time

**Figure 3:**
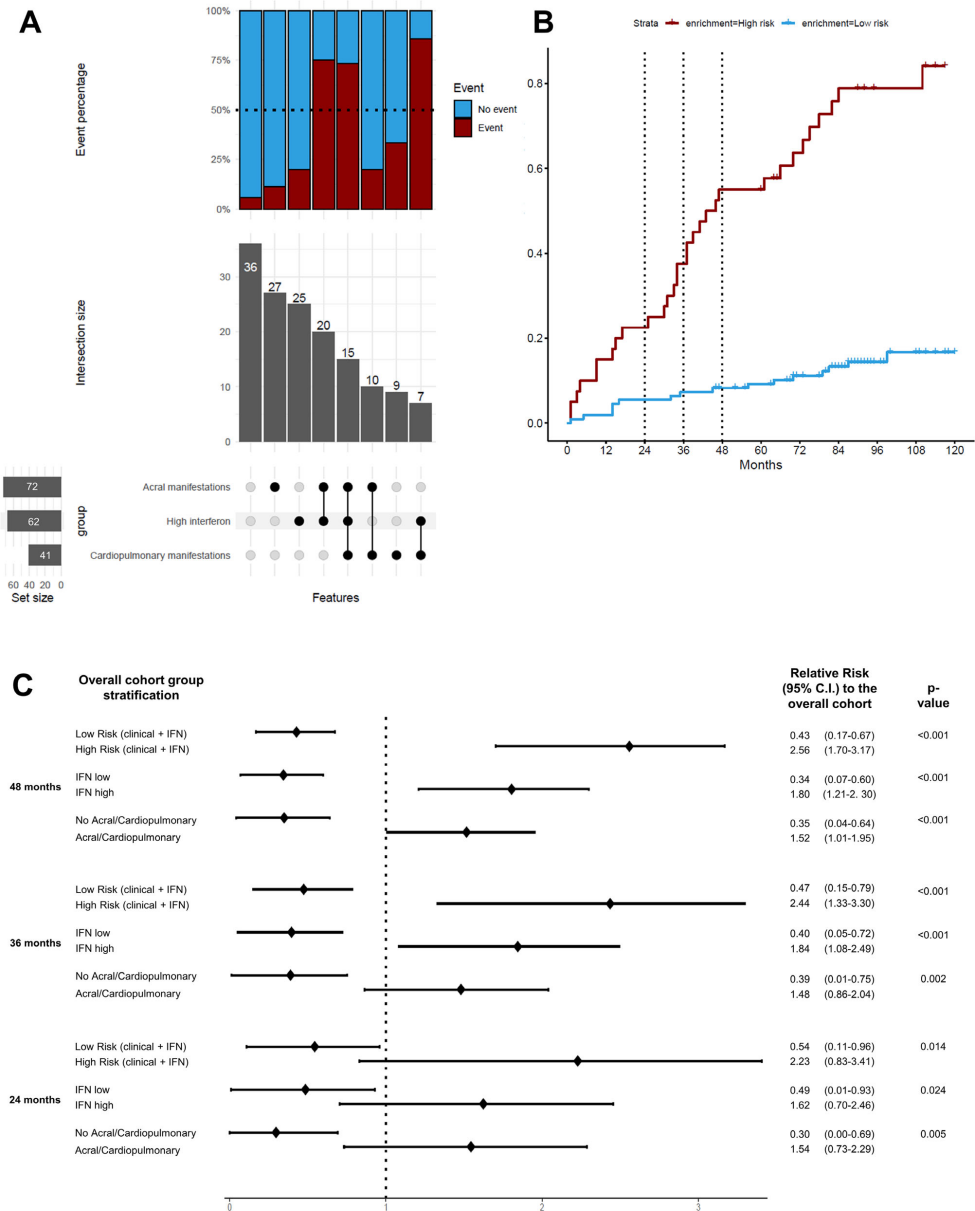
Combinations of clinical and serum IFN score groups. (A) Upset plots of clinical and serological subgroups with relative numerosity, prevalence, and interaction size. (B) Kaplan‐Meier curves for cumulative events for patients in the high‐risk and low‐risk groups for 120‐month follow‐up. (C) Forest plot for three time points relevant for clinical trials showing the relative risk for time to clinical worsening compared with the overall cohort for different stratification groups. The dotted line on 1 represents the risk of the unselected population for that relative time point. C.I., confidence interval; IFN, interferon.

Based on the significant predictors in the multivariate CPH regression (namely, IFN score, mRSS, DU disease, ILD, and PAH), we built upset plots depicting the prevalence and clinical outcome of these features alone or in combination in our population (Figure 3A). To allow a meaningful visualization of subgroups, patients fulfilling the mRSS >4 or baseline DU disease criteria were grouped as having “acral involvement,” whereas patients with PAH or ILD at baseline were classified as presenting “cardiopulmonary involvement.” Patients meeting the criteria for both acral and cardiopulmonary involvements were recognized as having dual involvement. Conversely, patients showing no clinical involvement at baseline were categorized as having none. The four clinical groups were further stratified based on IFN high or low categories leading to eight strata. The upset plot distinctly highlighted that only the strata characterized by high IFN in combination with acral and/or cardiopulmonary manifestations notably exceeded the 50% threshold for event occurrence. In contrast, other patient subsets fell below the 50% threshold, indicating a lower incidence of events. Notably, among the incident cases without acral or cardiopulmonary manifestations at baseline, 2 of 36 low IFN patients developed the outcome (5.6%) compared with 5 of 25 high IFN patients (20.0%), reflecting a nearly four‐fold higher incidence rate despite the low numbers. Accordingly, when comparing these two groups without baseline clinical manifestations, the restricted mean TTCW was shorter for the high IFN group compared with the low IFN group (104.5 months, 95% CI 92.0–117.1 vs 116.7 months, 95% CI 112.0–121.3) with a between‐group difference of −12.1 months (95% CI −25.5 to 1.3; *P* = 0.076).

On this basis, patients fulfilling at least one clinical criterion (acral and/or cardiopulmonary) and the serologic criterion of high IFN were classified as being “high risk,” whereas those not fulfilling both criteria were deemed to be “low risk.” The 10‐year event‐free survival curve of these two groups highlighted a major difference in restricted mean TTCW (55.7 months [95% CI 43.6–67.9] vs 105.8 months [95% CI 100.4–111.3] for high risk vs low risk, respectively, *P* < 0.001) (Figure 3B; Supplementary Table 5). Forest plots of the RR of each group at 24, 36, and 48 months relative to the overall cohort (Figure 3C) indicated that at 24 months the RR for the IFN high group is 1.62 (95% CI 1.07–2.48), whereas for the IFN low group it is 1.22 (95% CI 0.81–1.83). The high‐risk (at least one clinical criterion and a high IFN score) group's RR is 2.20 (95% CI 1.84–2.66), contrasting with the low‐risk group at 1.10 (95% CI 0.99–1.21). Patients with acral and/or cardiopulmonary manifestations had an RR of 1.56 (95% CI 1.11–2.01) against 0.90 (95% CI 0.75–1.05) for those without. At 36 months, the RR of the IFN high group was 2.35 (95% CI 1.88–2.92), and the IFN low one was 1.31 (95% CI 0.98–1.74). The high‐risk group's RR was 2.75 (95% CI 2.31–3.19), whereas the low‐risk group remained relatively stable at 1.15 (95% CI 1.02–1.28). The presence of acral and/or cardiopulmonary manifestations resulted in an RR of 1.75 (95% CI 1.39–2.11), with the absence of these manifestations showing an RR of 0.95 (95% CI 0.80–1.10). At 48 months, data consolidation occurs with increases in RR for long‐term follow‐ups in the high‐risk and IFN high groups, indicating a sustained risk over time.

## DISCUSSION

Our study shows for the first time that patients with lcSSc show a high IFN activation in their serum in a proportion even higher than the one observed in dcSSc. Given the effect of standard immune suppression on serum IFN score shown in the Scleroderma Lung Study II cohort, it is plausible to speculate that the increased proportion observed in patients with lcSSc may be driven by a lower prevalence of immune‐suppressant medications in this cohort (17.4% vs 70% of dcSSc).^24^ Herein we show that patients with high IFN activity at baseline have a worse clinical outcome as assessed by an increased proportion of patients meeting the combined event (57% vs 12%) and a shorter restricted mean TTCW (74.4 [95% CI 70.1–79.3] months in IFN high vs 110.6 [95% CI 107.2–114.0] months in IFN low).

It is intriguing to observe that despite this negative prognostic value, there was no difference at baseline in the prevalence of clinically meaningful organ manifestations. This observation supports the notion that type I IFN signature may reflect biologic disease activity before clinically detectable damage.^25^ Although this was an analysis of a prevalent cohort with highly variable disease duration at time of enrollment, we did not observe a significant difference in disease duration across IFN groups or event groups. The observation that disease duration did not differ in patients with or without events may support that the concept of accumulation of disease damage over time, true for most inflammatory conditions, may not entirely apply to lcSSc and/or may be mitigated by survival bias. In this context, the value of a specific type of disease activity (eg, type I IFN) may play a more important role than disease duration. Longitudinal studies are ongoing to determine the effect of disease duration or immunomodulatory treatments on type I IFN score and how this can modulate its value in predicting for clinically meaningful events.

Our results, identifying the baseline presence of ILD, PAH, and more severe skin involvement as predictive of disease complications, align with the established literature regarding prognostic factors in SSc, affirming known associations between baseline organ involvement, namely ILD, PAH, and skin fibrosis, and SSc‐related events.^7^, ^26^, ^27^ These associations likely stem from the intrinsic risk of SSc‐related mortality inherent in these conditions, specifically with regard to PAH and ILD, but they also potentially signify a more severe disease phenotype that elevates the risk of other organ failures and clinically significant events. Additionally, consistent with prior multicenter studies,^28^, ^29^, ^30^ a history of DUs in our cohort increased the risk of severe vascular events, worse disease progression, and survival outcomes. The MINIMISE composite endpoint of disease progression includes change in mRSS. We consider this to be an important and novel aspect of our work but appreciate that the threshold for clinically meaningful progression is extrapolated from previous studies of dcSSc. This is a limitation, and future work examining other independent SSc cohorts will determine if this threshold may be adjusted for application to lcSSc. Using similar clinical features, the revised European Scleroderma Trials and Research Activity Index (EUSTAR‐AI) has been proposed as a score to enrich for poor outcome in dcSSc.^31^ In our cohort, only 7 of 149 patients had EUSTAR‐AI scores above 2.5 (ie, active disease), as one would expect in the lc subset; nonetheless, the IFN score did correlate with EUSTAR‐AI, although weakly (Spearman ρ = 0.230; *P* = 0.006).

Our findings further support the significance of antibody profiles known to exhibit both positive and negative prognostic implications. Specifically, the observed lower incidence of Morbi‐mortality events in patients solely positive for ACA resembles trends from other incident SSc cohorts.^7^ Notably, we observed a numerically higher proportion of patients with low IFN score presenting an isolated ACA positivity (45% vs 33%) with a trend toward significance. This latter observation may explain the loss of significance of ACA positivity in multivariate analysis with IFN score. Further, our investigation revealed a significantly higher prevalence of anti‐Ro52 antibodies in the IFN high group, suggesting a potential relationship between autoantibody profiles and differential IFN expression. The prevalence of the Ro‐52 autoantibody in our cohort mirrors findings from significant multicentric studies,^32^ with its positivity previously linked to markedly worse survival rates, PAH, and ILD progression.^33^, ^34^ Nonetheless, in our lcSSc cohort, anti‐Ro52 failed to confer a significantly increased risk of events, likely because of the inclusion of a small subset of patients with overlapping Sjögren disease. Notably, the presence of another autoimmune disease overlap has been shown to confer a milder phenotype in SSc,^35^ which may mask the potential impact of anti‐Ro52 antibodies on disease severity.

Furthermore, we showed that the integration of established clinical features such as ILD, PAH, and mRSS with the IFN score improved the prediction of trial‐relevant events. This fosters a paradigm shift toward personalized medicine in lcSSc trials. In fact, this composite model not only enables the identification of patients at higher risk of specific complications but also facilitates the evaluation of therapeutic interventions tailored to individual risk profiles.^36^, ^37^ Lastly, the inclusion of antibody profiles, with anti‐Ro52 indicating a higher risk of adverse outcomes and, even more so, the exclusion of the isolated ACA‐positive profile showcasing a protective association, could refine even further lcSSc trial cohorts, enabling a more effective evaluation of therapeutic efficacy in intervention studies.

Our study retains inherent limitations because of its retrospective nature, which is primarily susceptible to information bias and nonparticipation bias, the latter potentially censoring patients with milder phenotypes and thus underpowering the analysis. In the attempt to mitigate this limitation, we have performed an analysis on the patients with complete 5‐year follow‐up data available, and we have observed a very similar effect on type I IFN score. An additional limitation is that although our sample size was calculated for detecting differences in event‐free survival between lcSSc groups, the validation of our proposed multivariable model metrics necessitates a larger confirmatory cohort, as our current numbers of events precluded cross‐validation approaches. Future, larger‐scale, multicentric studies will be instrumental in refining potential patient‐selection tools.

Another aspect of interest regarding the utility of IFN score lies in understanding its temporal dynamics. Given its demonstrated dysregulation across various autoimmune conditions,^18^ elucidating its temporal kinetics is pivotal to portray IFN score as a dynamic biomarker, revealing SSc disease flares and progression, or potentially establishing this biomarker as a stable pathogenetic signature, akin to antibody profiles. Assessing the longitudinal change in IFN score over time is therefore warranted for identifying a time‐dependent association with the onset of clinically apparent organ damage. In this sense, comparing the trajectories of IFN scores in patients with differing outcomes over time could be very useful to understand the effect of molecular heterogeneity in a longitudinal setting. Moreover, it remains to be assessed whether, similarly to what has been observed in the Scleroderma Lung Study II,^24^ serum IFN score may change following immune suppression in lcSSc. Longitudinal studies are ongoing to determine the effect of disease duration or immunomodulatory treatments on type I IFN score and how this can modulate its value in predicting for clinically meaningful events.

In conclusion, our data demonstrate that type I IFN activation is common in SSc, extending to both cutaneous subsets. Our data also demonstrate that the IFN score may aid in the assessment of disease activity and in enrichment for a higher probability of clinically meaningful events over time in lcSSc trials.

## AUTHOR CONTRIBUTIONS

All authors contributed to at least one of the following manuscript preparation roles: conceptualization AND/OR methodology, software, investigation, formal analysis, data curation, visualization, and validation AND drafting or reviewing/editing the final draft. As corresponding author, Drs Del Galdo and Denton confirm that all authors have provided the final approval of the version to be published, and take responsibility for the affirmations regarding article submission (eg, not under consideration by another journal), the integrity of the data presented, and the statements regarding compliance with institutional review board/Declaration of Helsinki requirements.

## Supporting information


**Disclosure Form**:

**Appendix S1:** Supporting Information
