# Supplementary material for: Serum Type I Interferon Score for Prediction of Clinically Meaningful Disease Progression in Limited Cutaneous Systemic Sclerosis
Source: Arthritis Rheumatol. 2025 Mar 3;77(7):929–41. doi: 10.1002/art.43120 (PMC12209744; doi:10.1002/art.43120)
Supplement: Supplementary file 2 — Appendix S1: Supporting Information [file ART-77-929-s001.docx]

Appendix 1.

**
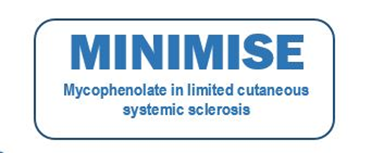
Mycophenolate in limited cutaneous systemic sclerosis (MINIMISE) clinical trial endpoint definition of “time to clinical worsening”.**

**Chief Investigator: Professor Christopher P. Denton**

**Registration: ClinicalTrials.gov NCT04927390**

**EudraCT 2019-004139-21**

Primary efficacy endpoint is an event-driven analysis comparing the randomly allocated mycophenolate mofetil and no immunosuppression study arms.

- Time to clinical worsening of lcSSc defined as “development of new clinically significant pulmonary, cardiac, renal or gastro-intestinal involvement; or severe digital vasculopathy; or significant increase in modified Rodnan skin score (MRSS).”

The clinical worsening composite endpoint consists of the following:

- New lung fibrosis on HRCT with FVC< 70%. This will require definite CT appearance of lung fibrosis with at least 5% of lung involved and FVC less than 70% predicted with technically acceptable spirometry.
- Deterioration of established lung fibrosis (composite categorical decline (CCD) defined by reduction in FVC by at least 10% or FVC 5-9% with DLco 15% fall). Cases will all have definite lung fibrosis affecting at least 5% of lung fields and changes in lung function will be interpreted only with technically satisfactory measurement. Changes will be absolute % predicted.
- Significant progression of modified Rodnan skin score (MRSS increase of at least 5 units and 25% increase from baseline). Assessment by an assessor proficient in MRSS.
- Hemodynamically significant cardiac complication related to SSc defined by systolic ejection fraction less than 45% or large pericardial effusion impairing cardiac function or arrhythmia requiring anti- arrhythmic therapy (cardioversion, medical or device) or need for a cardiac pacemaker.
- Scleroderma renal crisis as defined by the new proposed classification criteria.
- Confirmed new diagnosis of pulmonary hypertension by RHC according to the new 2019 expert definition (mPAP above 20 mm Hg, PVR above 3 Wood Units).
- SSc related GI disease requiring enteral nutritional supplementation for more than 3 weeks or any parenteral feeding or admission over 72 hours for intestinal obstruction or pseudo-obstruction.
- Severe digital vasculopathy (defined as gangrene, amputation, osteomyelitis, or septic arthritis as assessed by the investigator and confirmed by imaging or culture).
- Scleroderma-related Mortality.

Supplementary table 1. Descriptive table showing events following the first one and not included in the main analysis by patient. The Interferon group for each patient experiencing the MINIMSE endpoint is reported.

| **Patient ID** | **First Event** | **Second Event** | **Third Event** | **IFN Group** |
| --- | --- | --- | --- | --- |
| 1 | Cardiac | - | - | High |
| 2 | Cardiac | - | - | High |
| 3 | Cardiac | - | - | High |
| 4 | Cardiac | - | - | High |
| 5 | Cardiac | Progression of Existing or New Onset ILD | Mortality | Low |
| 6 | Cardiac | - | - | Low |
| 7 | GI | Mortality | - | High |
| 8 | GI | - | - | High |
| 9 | GI | - | - | High |
| 10 | GI | Mortality | - | High |
| 11 | Mortality | - | - | High |
| 12 | Mortality | - | - | High |
| 13 | Mortality | - | - | Low |
| 14 | Mortality | - | - | High |
| 15 | Mortality | - | - | High |
| 16 | Mortality | - | - | High |
| 17 | Mortality | - | - | High |
| 18 | Mortality | - | - | High |
| 19 | Mortality | - | - | High |
| 20 | PAH | Mortality | - | High |
| 21 | PAH | - | - | High |
| 22 | PAH | Mortality | - | High |
| 23 | PAH | - | - | High |
| 24 | PAH | - | - | High |
| 25 | PAH | Cardiac | - | High |
| 26 | PAH | - | - | Low |
| 27 | PAH | - | - | High |
| 28 | PAH | Cardiac | Skin (mRSS) | Low |
| 29 | PAH | - | - | Low |
| 30 | PAH | Severe Digital Vasculopathy | - | High |
| 31 | PAH | Progression of Existing or New Onset ILD | - | High |
| 32 | PAH | Progression of Existing or New Onset ILD | Skin (mRSS) | Low |
| 33 | Progression of Existing or New Onset ILD | - | - | High |
| 34 | Progression of Existing or New Onset ILD | Mortality | - | High |
| 35 | Progression of Existing or New Onset ILD | Mortality | - | Low |
| 36 | Progression of Existing or New Onset ILD | - | - | Low |
| 37 | Progression of Existing or New Onset ILD | - | - | High |
| 38 | Progression of Existing or New Onset ILD | PAH | - | High |
| 39 | Renal Crisis | Progression of Existing or New Onset ILD | - | High |
| 40 | Severe Digital Vasculopathy | - | - | High |
| 41 | Severe Digital Vasculopathy | - | - | Low |
| 42 | Severe Digital Vasculopathy | Severe Digital Vasculopathy | - | High |
| 43 | Skin (mRSS) | Cardiac | - | High |
| 44 | Skin (mRSS) | Cardiac | - | High |
| 45 | Skin (mRSS) | - | - | High |
| 46 | Skin (mRSS) | - | - | High |
| 47 | Skin (mRSS) | - | - | High |

Supplementary table 2. Time to clinical worsening (TTCW) table of the overall cohort at 12, 24, 36, 60, and 120 months.

| **Time** | **Number at risk** | **Number of events** | **Survival** | **Std. error** | **Lower 95% CI** | **Upper 95% CI** |
| --- | --- | --- | --- | --- | --- | --- |
| 12 | 141 | 8 | 94.6% | 1.85 | 91.1% | 98.3% |
| 24 | 134 | 7 | 89.9% | 2.47 | 85.2% | 94.9% |
| 36 | 126 | 8 | 84.6% | 2.96 | 79.0% | 90.6% |
| 60 | 113 | 9 | 78.5% | 3.37 | 72.2% | 85.4% |
| 120 | 25 | 15 | 65.1% | 4.43 | 57.0% | 74.4% |

Supplementary figure 1. Incident-case/dynamic-control time-dependent ROC curve at 3 timepoints relevant for clinical trials (12, 24, and 36 months) for the multivariate Cox proportional hazard models built with clinical predictors and IFN score continuous (A) or categorical (B), with IFN score alone (C), and clinical predictors alone (D).


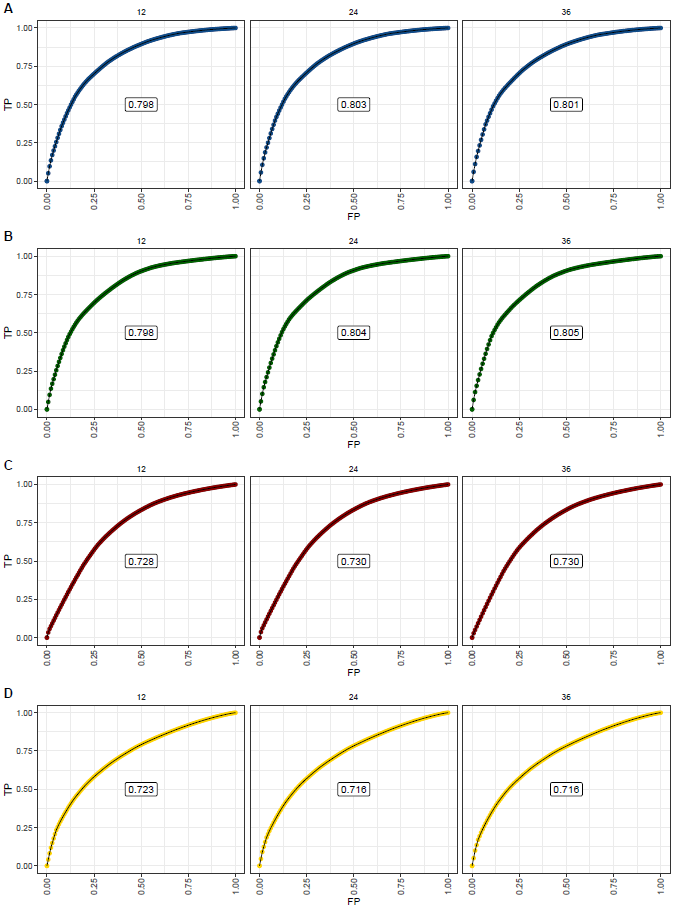
Supplementary figure 2. Kaplan-Meier curves showing the 10-year Morbi-mortality event cumulative incidence probability. Interferon “High” (red) and “Low” (light blue) are shown. Two-sample t-test applied to the restricted mean survival times). Dotted line represents the restricted mean event-free survival probability of IFN High subgroup.


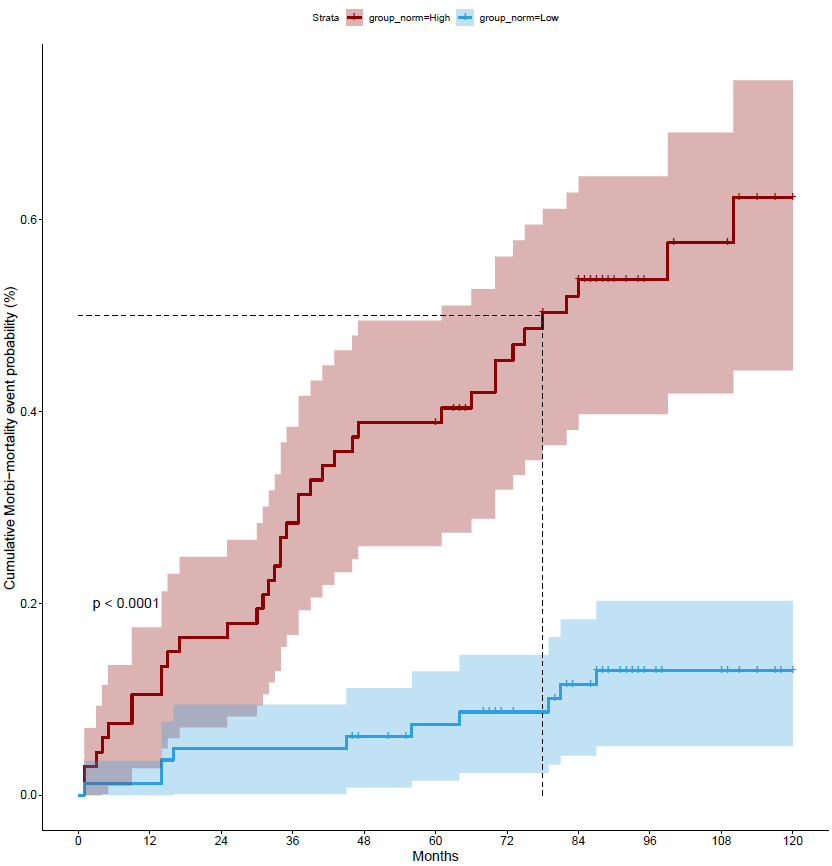


|  | **Time** | **Number at risk** | **Events** | **Event probability** | **Lower CI** | **Upper CI** | **Standard error** |
| --- | --- | --- | --- | --- | --- | --- | --- |
| **“Low” IFN** | 12 | 81 | 1 | 1.2% | 0.0% | 3.6% | 1.2% |
|  | 24 | 78 | 3 | 4.9% | 0.1% | 9.4% | 2.4% |
|  | 36 | 78 | 0 | 4.9% | 0.1% | 9.4% | 2.4% |
|  | 48 | 75 | 1 | 6.1% | 0.8% | 11.1% | 2.6% |
|  | 60 | 72 | 1 | 7.4% | 1.5% | 12.9% | 2.9% |
|  | 120 | 20 | 4 | 13.0% | 5.1% | 20.2% | 3.9% |
| **“High” IFN** | 12 | 60 | 7 | 10.4% | 2.8% | 17.5% | 3.7% |
|  | 24 | 56 | 4 | 16.4% | 7.1% | 24.8% | 4.5% |
|  | 36 | 48 | 8 | 28.4% | 16.7% | 38.4% | 5.5% |
|  | 48 | 41 | 7 | 38.8% | 26.0% | 49.4% | 6.0% |
|  | 60 | 41 | 0 | 38.8% | 26.0% | 49.4% | 6.0% |
|  | 120 | 5 | 11 | 62.3% | 44.3% | 74.5% | 7.5% |

Supplementary table 3. Event table for patients in the Interferon “High” and “Low” subgroups for 120-month follow up.

Supplementary figure 3. Kaplan-Meier curves showing the 5-year Morbi-mortality event incidence probability in IFN “High” (red) and “Low” (light blue) patients using only non-censored 5-year follow ups (two-sample t-test applied to the restricted mean survival times).


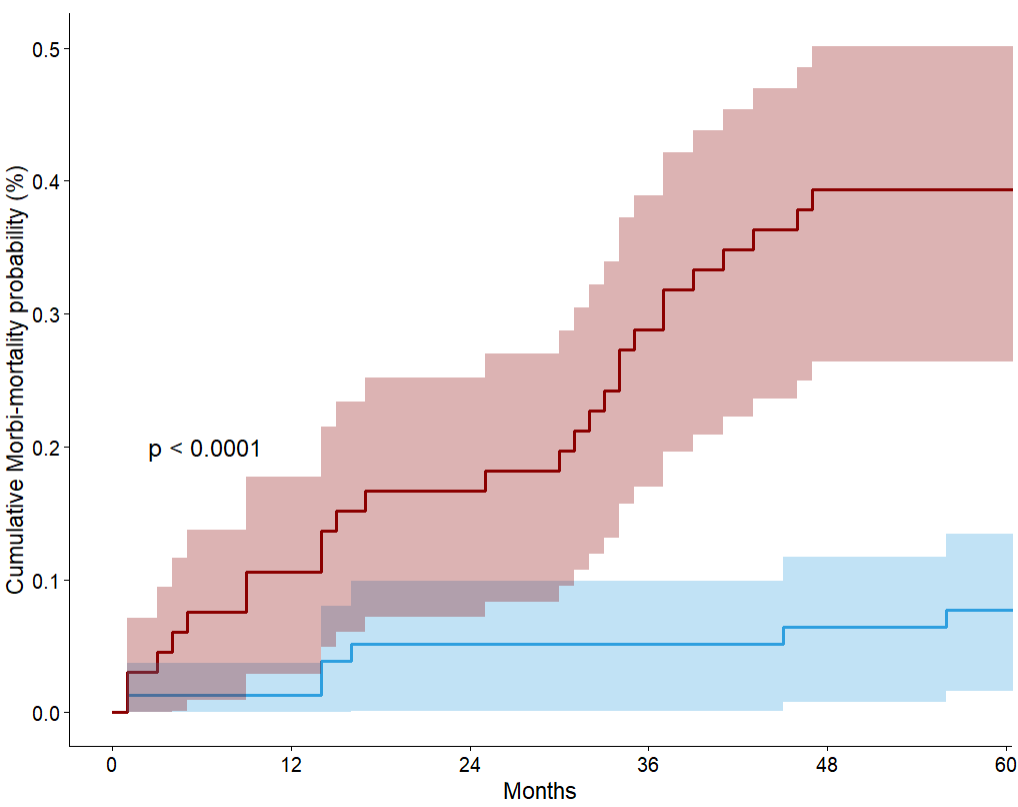


Supplementary table 4. Event table for patients in the Interferon (IFN) “High” and “Low” subgroups using only non-censored 5-year follow ups.

|  | **Time** | **Number at risk** | **Events** | **Event probability** | **Lower CI** | **Upper CI** | **Standard error** |
| --- | --- | --- | --- | --- | --- | --- | --- |
| **“Low”**  **IFN** | 12 | 77 | 1 | 1.3% | 0.0% | 3.7% | 1.3% |
|  | 24 | 74 | 3 | 5.1% | 0.1% | 9.9% | 2.5% |
|  | 36 | 74 | 0 | 5.1% | 0.1% | 9.9% | 2.5% |
|  | 48 | 73 | 1 | 6.4% | 0.8% | 11.7% | 2.8% |
|  | 60 | 72 | 1 | 7.7% | 1.6% | 13.4% | 3.0% |
| **“High”**  **IFN** | 12 | 59 | 7 | 10.6% | 2.9% | 17.7% | 3.8% |
|  | 24 | 55 | 4 | 16.7% | 7.2% | 25.2% | 4.6% |
|  | 36 | 47 | 8 | 28.8% | 17.0% | 38.9% | 5.6% |
|  | 48 | 40 | 7 | 39.4% | 26.4% | 50.1% | 6.0% |
|  | 60 | 40 | 0 | 39.4% | 26.4% | 50.1% | 6.0% |

Supplementary table 5. Event table for patients in the “High risk” and “Low risk” groups at 12, 24, 36, 48, 60, and 120-month follow up, after combination of clinical features (namely acral involvement, cardiopulmonary involvement, dual involvement, and no involvement) and Serum Interferon (IFN) score groups.

|  | **Time** | **Number at risk** | **Events** | **Event probability** | **Lower CI** | **Upper CI** | **Standard error** |
| --- | --- | --- | --- | --- | --- | --- | --- |
| **High risk** | 12 | 34 | 6 | 15.0% | 3.2% | 25.4% | 5.6% |
|  | 24 | 31 | 3 | 22.5% | 8.4% | 34.4% | 6.6% |
|  | 36 | 25 | 6 | 37.5% | 20.5% | 50.8% | 7.7% |
|  | 48 | 18 | 7 | 55.0% | 36.6% | 68.1% | 7.9% |
|  | 60 | 18 | 0 | 55.0% | 36.6% | 68.1% | 7.9% |
| **Low risk** | 12 | 107 | 2 | 1.8% | 0.0% | 4.3% | 1.3% |
|  | 24 | 103 | 4 | 5.5% | 1.1% | 9.7% | 2.2% |
|  | 36 | 101 | 2 | 7.3% | 2.3% | 12.1% | 2.5% |
|  | 48 | 98 | 1 | 8.3% | 2.9% | 13.3% | 2.6% |
|  | 60 | 95 | 1 | 9.2% | 3.6% | 14.5% | 2.8% |
|  | 120 | 25 | 6 | 16.7% | 8.4% | 24.3% | 4.1% |
